# Acute Pulmonary Embolism in a Healthy Male With Elevated Lipoprotein(a)

**DOI:** 10.7759/cureus.8005

**Published:** 2020-05-07

**Authors:** Subhankar Samal, Namrata Singhania, Saurabh Bansal, Anil Singh

**Affiliations:** 1 Internal Medicine, Ascension Health, Milwaukee, USA; 2 Hospital Medicine, Mount Carmel Hospital, Columbus, USA; 3 Internal Medicine, University of Illinois at Peoria, Peoria, USA; 4 Hospital Medicine, Geisinger Community Medical Center, Scranton, USA

**Keywords:** pulmonary embolism, lipoprotein (a)

## Abstract

Elevated lipoprotein(a) [Lp(a)] is a well-known risk factor for cardiovascular disease. Its role in venous thromboembolism (VTE) is unclear. We present a case of a healthy man presenting with bilateral pulmonary embolism and investigations suggested elevated Lp(a) being the only risk factor. In this case report, we would like to emphasize the role of Lp(a) in VTE without any other clear predisposing factors.

## Introduction

Acute pulmonary embolism (PE) is one of the common types of venous thromboembolism (VTE). It can be due to genetic or acquired causes. Elevated lipoprotein(a) [Lp(a)] has been associated with an increased cardiovascular risk [1]. There have been some cross-sectional studies proving the association of Lp(a) with VTE, but data are still controversial. There are no randomized controlled trials to prove this association. We, herein, present a case of an otherwise healthy young male with acute PE who has no genetic or acquired risk factors for VTE in the workup except elevated Lp(a).

## Case presentation

A 53-year-old healthy Caucasian male with no significant past medical history presented to the hospital with a three-week history of worsening productive cough with minimal hemoptysis, shortness of breath and chest pain. He was physically active and runs regularly. He was prescribed ciprofloxacin for pneumonia two weeks prior to admission with no improvement in symptoms. The patient denied any recent sick contacts, smoking and long-distance road or air travel. He did not have any history of significant weight loss. He was afebrile, heart rate was 65 beats/minute, respiratory rate was 18/minute, blood pressure was 124/64 mmHg and oxygen saturation was 95% on room air. His lung sounds were clear to auscultation. Rest of the examination was unremarkable.

**Figure 1:**
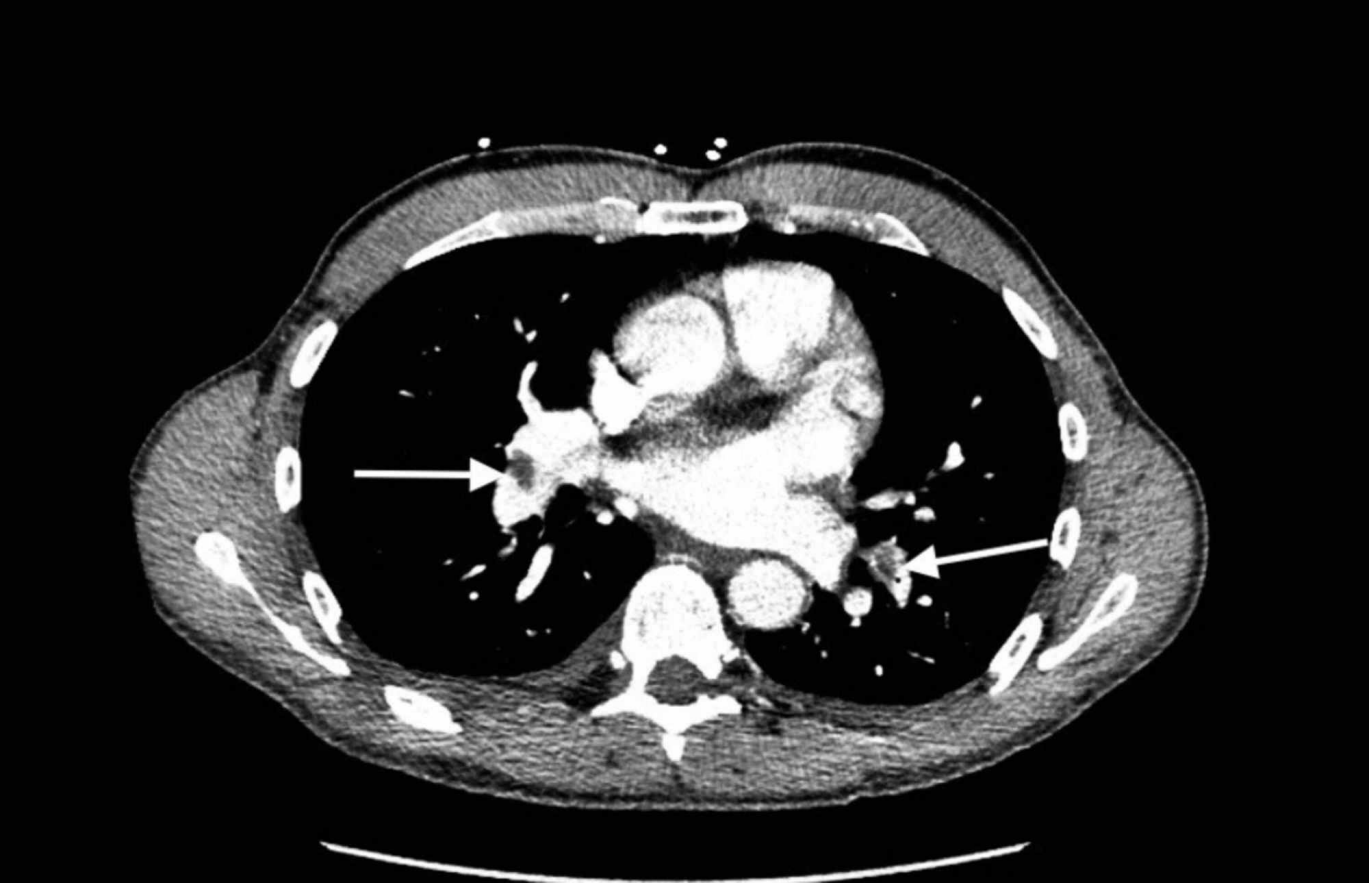
Bilateral pulmonary embolism (PE) CT scan of the chest with contrast revealed bilateral segmental and subsegmental PE within the bilateral lower lobes and right middle lobe.

Laboratory indices including complete blood count with differential, basic metabolic panel, troponin I and b-type natriuretic peptide levels were normal. CT scan of the chest with contrast revealed bilateral segmental and subsegmental PE within the bilateral lower lobes and right middle lobe (Figure 1).

Ultrasound of bilateral lower extremities did not reveal deep vein thrombosis. Transthoracic echocardiogram showed an ejection fraction of 55%-60%, no flattening of septum and no valvular abnormality. The patient was started on heparin infusion, and hematology was consulted. Hypercoagulable workup was ordered to look for cause of PE in this otherwise healthy male. No evidence of lupus anticoagulant and antiphospholipid antibody were detected in the serum. Antithrombin assay was 118% (normal 80-129), protein C activity was 114% (normal 77-133), protein S activity was 81% (normal 70-162) and activated protein C resistance was 2.5% (normal 2.2-4). Lp(a) level was significantly elevated at 71 mg/dL (normal <29). Homocysteine level was normal at 8.61 µmol/L (normal <16.19). The patient had screening colonoscopy two years prior, and biopsy was negative for malignancy. The patient was discharged home on apixaban, and at follow-up visit has recovered well with residual symptoms.

## Discussion

Acute PE is a common form of VTE that can sometimes cause hemodynamic instability when massive and can be fatal. Its incidence is higher in males compared with females (56 versus 48 per 100,000, respectively). The risk factors of PE can be classified as inherited (genetic) or acquired. The common inherited factors are factor V Leiden and prothrombin gene mutation, while the common acquired factors are recent surgery, malignancy, heavy smoking, immobilization and hormone therapy.

Lp(a) is a known risk factor for atherosclerosis, premature coronary artery disease, stroke and peripheral artery disease, but its role in VTE is unclear. Apolipoprotein(a) [Apo(a)] is the protein component of Lp(a), which is homologous to plasminogen. Therefore, Lp(a) possesses antifibrinolytic and prothrombotic properties [2]. Serum levels of Lp(a) are determined by genetic variation in the LPA gene encoding for Apo(a), including the kringle-IV type 2 (KIV-2) size polymorphism [3]. Sticchi et al. have reported that variation in KIV-2 significantly and independently predisposes to VTE [3].

Most of the studies showing the association between Lp(a) and VTE was cross-sectional and prospective trails are lacking. In a systematic review by Dentali et al., Lp(a) was significantly associated with an increased risk of VTE (odds ratio: 1.56, 95% confidence interval: 1.36, 1.79; 10 studies, 13,541 patients) [4]. The cut-off level used to define elevated Lp(a) was 30 mg/dL. A case-control study of Chinese patients showed an 10-fold increase in VTE with elevated Lp(a) >30 mg/dL [5]. Similarly, Lp(a) >30 mg/dL was found to be an independent risk factor (4.5-fold higher risk) for the first VTE in children as well [6]. Anticoagulation is the recommended treatment for VTE, but there is no published recommendation on the duration of treatment in the patients with elevated Lp(a). With its proven association with elevated cardiovascular risk, we strongly recommend these patients to follow up closely with their primary care physician or cardiology.

## Conclusions

Although it is hard to associate a causality based on this case report, we believe that elevated Lp(a) level was associated with PE in our patient and suggest checking Lp(a) level in cases of VTE with no other identifiable risk factors. Prospective trials are needed to prove this association.
